# Potential risk assessment for safe driving of autonomous vehicles under occluded vision

**DOI:** 10.1038/s41598-022-08810-z

**Published:** 2022-03-23

**Authors:** Denggui Wang, Weiping Fu, Qingyuan Song, Jincao Zhou

**Affiliations:** 1 School of Mechanical and Precision Instrument Engineering, Xi’an University of Technology, Xi’an, China; 2 School of Engineering, Xi’an International University, Xi’an, China

**Keywords:** Mechanical engineering, Computer science

## Abstract

This study aimed to explore how autonomous vehicles can predict potential risks and efficiently pass through the dangerous interaction areas in the face of occluded scenes or limited visual scope. First, a Dynamic Bayesian Network based model for real-time assessment of potential risks is proposed, which enables autonomous vehicles to observe the surrounding risk factors, and infer and quantify the potential risks at the visually occluded areas. The risk distance coefficient is established to integrate the perception interaction ability of traffic participants into the model. Second, the predicted potential risk is applied to vehicle motion planning. The vehicle movement is improved by adjusting the speed and heading angle control. Finally, a dynamic simulation platform is built to verify the proposed model in two specific scenarios of view occlusion. The model has been compared with the existing methods, the autonomous vehicles can accurately assess the potential danger of the occluded areas in real-time and can safely, comfortably, and effectively pass through the dangerous interaction areas.

## Introduction

The current autonomous driving technology still faces many problems, the safety problem of autonomous vehicles (AVs) has been given highly recognition and close attention by public^1^. For safety reasons, the safety officer must continuously monitor the autonomous driving system and cannot achieve fully automated driving^2^. The uncertainty and potential danger caused by the vision of the vehicle sensors are often the corner case in the safety problem of autonomous driving. The so-called "potential danger scene" refers to when the autonomous vehicle (AV) is driving in the current driving area, other traffic participant (TP) may enter the current driving area from the area where the visual field of the AV's optical sensor is blocked, which may lead to unpredictable conflicts^3,4^. The sensor range is limited, especially on some urban roads due to occlusions caused by continuously parked cars or buildings. The AV does not respond in time to the sudden appearance of pedestrians in the driving area of the vehicle, resulting in traffic accidents^5^. In recent years, the uncertain potential risk caused by visual field occlusion has attracted the attention of the academic community. This paper proposes a real-time risk assessment algorithm applied to urban road scenes with intermittent occlusion, focusing on quantifying the risk in the environment. The method is suitable for the uncertainty of the traffic environment and the movement of TPs, and uses probability to quantify the potential risk in the dangerous area.

The remainder of this paper is arranged as follows: The rest of this part reviews the current work related to risk assessment for autonomous driving in scenarios with occlusion or limited visibility; In “Method”, proposes the potential risk assessment model in detail and shows how the AV applies potential risks in motion planning; Then, validates the effectiveness of the proposed model through simulation tests and results analysis; Last, concludes and discusses the future direction of the present work.

Lefèver et al. Related research performed a detailed review of motion prediction and risk assessment^6–8^. Generally, there are two main types of risk assessment, the deterministic method and the probabilistic method. The deterministic approach is a binary prediction that only estimates whether potential collisions will occur^9–12^. Probabilistic methods are usually applicable to the temporal and spatial relationship between vehicles, even the uncertainty of input data, and probabilistic descriptions to model the risk level^8,9,13–17^. This paper focuses on predicting accident probability (potential risk) caused by visual field occlusion. The potential risk can be solved by expanding the field of vision^18,19^ or by introducing prediction individuals and potential obstacles (also known as phantom or virtual objects)^14,20–24^. These strategies become very difficult to address in dynamic occlusion scenes. In recent motion planning, Partially Observable Markov Decision Process (POMDP) have optimized the behavior of AVs, thus reducing the collision risk caused by occlusion^25–28^. However, the calculation amount of the POMDP method increases exponentially with the number of states required for operation, which limits its applicability. To reduce the conservatism of vehicle operation, Schratter et al. activated an automatic emergency braking system (AEB) in a critical situation^29^. However, it does not to infer the sudden appearance of pedestrians according to the surrounding environment, and only uses the AEB method when pedestrians to suddenly rush out, the comfort of vehicles in the intermittent visual field occlusion scene needs to be verified. In the early stage, considering occlusion was used in the motion planning of mobile robots^18,30,31^, later, the risk assessment system of road vehicles considered the scene of occluded intersection. However, most of the above literature is limited to studying the interaction between vehicles in intersection occlusion scenes. At the same time, there is little literature on roadside occlusion scenes, especially in roadside intermittent occlusion complex scenes with more pedestrians. Recent works utilize forward reachability analysis to over-approximate potential occluded objects’ future occupancy based on the current sensor observations and generate collision-free trajectories by avoiding the entire forward reachable set (FRS) over the planning horizon^14,17,32^. However, this approach ignores the AVs’ future ability to respond to objects, which are currently occluded but may later become visible through new sensor observations.

Dynamic Bayesian networks (DBNs) are widely applied to reasoning problems with dynamic uncertainty^16,33–35^^.^ This paper uses the assessment method based on DBN to quantitative analysis the potential risk of visual field occlusion and combines qualitative knowledge, such as expert experience judgement, with quantitative knowledge. The primary contributions of this study are as follows: (1) A DBN-based probability model is created, with which the potential risks from visually occluded areas are quantified. (2) A distance and velocity-based risk distance coefficient model is proposed to represent TPs' perceptual interaction function. (3) Motion planning of AVs is improved in intermittently occluded scenarios based on the results of potential risk assessment.

## Method

**Figure 1:**
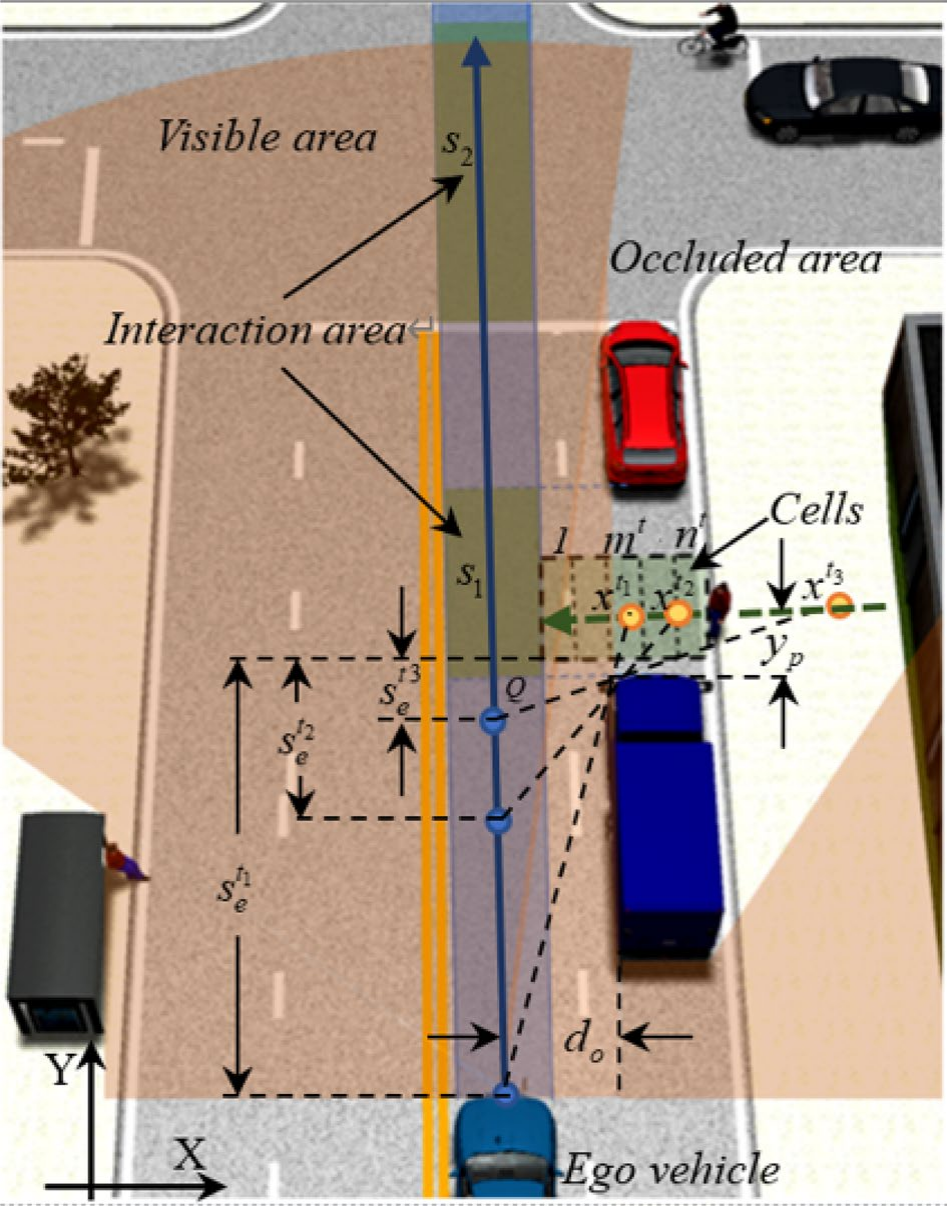
Schematic diagram of the model-the blue car is the ego vehicle, the truck and red car are occlusions, and the pedestrians, bicycles, black car are occluded TPs. Accidents may occur in interaction areas.

The potential risk $\gamma_{c}$ proposed in this paper refers to the probability or possibility of collision with TP, which came out of the occluded area all of a sudden, during AV passes through the interaction area. As expressed in $\gamma_{c} = [0,1]$, the potential risk varies with different traffic environments^36^. This paper takes the scene of visual field occlusion on one side of the AV as an example, as shown in Fig. 1, the occluded area may suddenly appear TPs (e.g. pedestrians passing through the clearance between vehicle and vehicle, bicycles or cars passing through the intersection), and the TP may enter the current driving area of the AV. Figure 1 shows common scenes of road and intersection with single occlusion or intermittent occlusion, such as pedestrian crossings, bus stops and intersections. The variables $v_{e}^{{t_{{}} }}$ and $s_{e}^{t}$ represent the speed of the AV and distance between the AV and the interaction area at time $t$ respectively. The variable $t_{c}$ represents the time required for the AV to pass through the interaction area which length is $s_{i}$, $t_{c} = s_{i} /v_{e}^{t}$. At the current speed, it can be predicted that the AV will reach the Q point of the interaction area after $t_{e} = s_{e}^{{t_{{}} }} /v_{e}^{{t_{{}} }}$ seconds. Whether the AV continues to pass through the interaction area at the current speed depends on the predicted potential risk $\gamma_{c}$ value within the time $t_{c}$. Let $v_{p}$ represent the average speed of the occluded TP in $\Delta t$ times, $\Delta d = v_{p} \cdot \Delta t$ indicates the distance traveled by traffic participants in $\Delta t$ times. At any time $t$, the occluded area is divided into $n^{t}$ cells along the X direction, $n^{t} = (t_{e} + t_{c} )/\Delta t$. Let $m^{t} = t_{e} /\Delta t$; when the occluded TP move forward with $v_{p}$, the TPs on the $n_{c} (n_{c} = n^{t} - m^{t} )$ cells will arrive at the risk interaction area in the $t_{e} \sim (t_{e} + t_{c} )$ period, resulting in potential risks to the AV. Using $\gamma_{c,i}^{t} \, (i \in m^{t} ,m^{t} + 1, \cdots ,n^{t} ) \,$ to represent the risk probability generated by cell $i$ at time $t$, the maximum value of $\gamma_{c,i}^{t} \,$ is recorded as $\gamma_{c}$:

1 $$ \gamma_{c} = \mathop {\arg \max }\limits_{{\gamma_{c,i}^{t} }} \left\{ {{\mathbb{R}}[\gamma_{c,i}^{t} \, |O^{t} ] \, (i \in [m^{t} ,n^{t} ])} \right\} $$

where $O^{t}$ represents the observed value of the AV around the interaction area at time $t$, which will be described in the later part.

As shown in Fig. 1, the distance between the AV and the interaction area at $t_{1}$ is $s_{e}^{{t_{1} }}$, and the visual range in the X direction of the interaction area is $x^{{t_{1} }}$. As the vehicle moves forward, the visual range gradually increases, and the visual range at $t_{2}$ and $t_{3}$ becomes $x^{{t_{2} }}$ and $x^{{t_{3} }}$ respectively. $y_{p}$ represents the minimum distance between the occlusion and the TPs, and $d_{o}$ represents the transverse distance between the centerline of the AV and the occlusion, so the visual range $x^{{t_{{}} }}$ of the AV in the X direction of the interaction area at time $t_{{}}$ as:

2 $$ x^{t} = d_{o} + \frac{{d_{o} \cdot y_{p} }}{{s_{e}^{t} }} = \frac{{d_{o} \cdot (s_{e}^{t} + y_{p} )}}{{s_{e}^{t} }} $$

Equation (2) indicates that the visible range $x^{t}$ at time $t_{{}}$ is related not only to $s_{e}^{t}$ but also to $y_{p}$ and $d_{o}$. When the vehicle is driving, it is as far away from obstacles as possible, which is in line with the human driving experience.

### Potential risk assessment model

**Figure 2:**
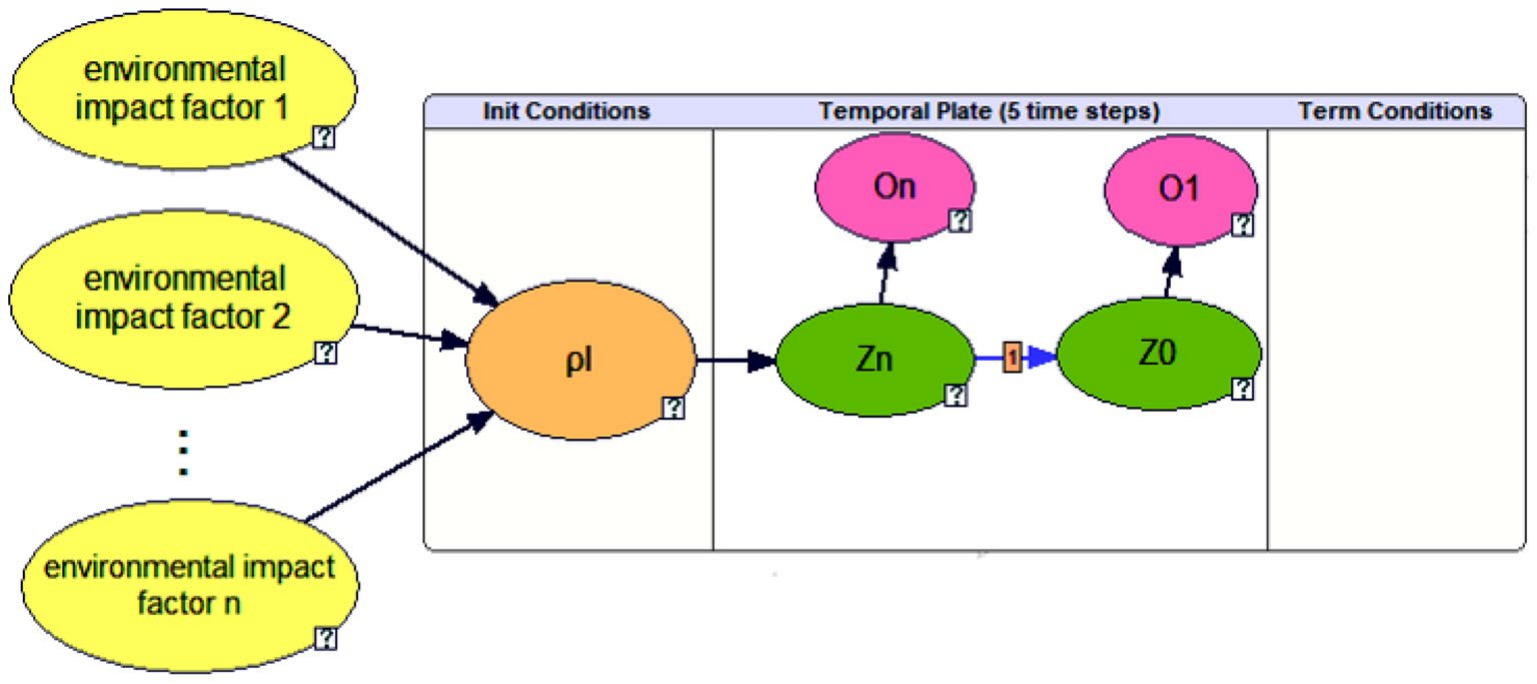
The structure of risk assessment model.

Simulate the principle of potential risk assessment by human drivers, the core idea of the proposed model is that the AV can inferring the potential risk probability of the occluded area by observing the surrounding environmental. To facilitate the description, pedestrians are the main TPs. The proposed risk assessment model based on DBN analysis is shown in Fig. 2, $\rho_{l}$ is used to represent pedestrians illegally crossing the road in the initial section (cell $n^{t}$) of the occluded area, its probability value $P(\rho_{l} )$ can be predicted and inferred by AVs observing the state of environmental impact factors (yellow nodes). $\rho_{l} = 1$ represents the event is occurrence, and $P(\rho_{l} = 1)$ is the occurrence probability, that is, the prior probability of risk assessment. Node "*Zi*" represents the event in which the road cell $i$ is or not occupied by pedestrians at time $t_{{}}$, and the node "*Oi*" represents the observation event of the AV to the road cell $i$. The AV infers the potential collision risk by observing whether pedestrians occupy the road, that is posteriori probability, $P(Z = 1|O)$. This paper uses GeNle software to help build Bayesian network. The reasoning mechanism of this software can perform fast and accurate causal reasoning.

It should be noted that the acquisition and measurement technology of variables is not the focus of this research, so it is not introduced in detail in this paper. We assume that the autonomous vehicle can accurately identify the surrounding traffic participants in real time by on-board sensors, and can also accurately measure and obtain various kinds of variables required by the model.

#### Prior probability $P(\rho_{l} = 1)$

In the occluded areas proposed above, the probability of pedestrians darting out $P(\rho_{l} = 1)$ is uncertain, and the probability varies with different factors, such as traffic environment and time. Determination of risk influencing factors is a crucial issue, which can be carried out by reviewing literature, consulting experts and analyzing historical data. In actual risk assessment, however, it is impossible to establish an assessment model that includes all indicators due to restrictions from the availability of data and the impact of indicators on assessment results. Akin et al. observed pedestrian crossing behavior near Michigan State University through previous video data and concluded that more than 90% of people would choose crosswalks in road sections without signal control^37^. In the study of pedestrian crossing behavior without signal lights in nine urban sections of Changsha in China^38^, 1275 groups of sample data show that 42.35% of pedestrians have "crossed the street illegally" on the main road. The proportion of pedestrians "crossing the street illegally" is 63.8% lower when there is a divider than when there is no divider, and the number of one-way lanes is 2, 3,4, the proportion is 36.7%, 50.3%, and 71.6% lower respectively than one-way lanes is 1. When pedestrians "cross the street illegally", the general headway on the road is H ≥ 2.7 s, and the vehicle speed range is V ≤ 7.78 M/s.

**Figure 3:**
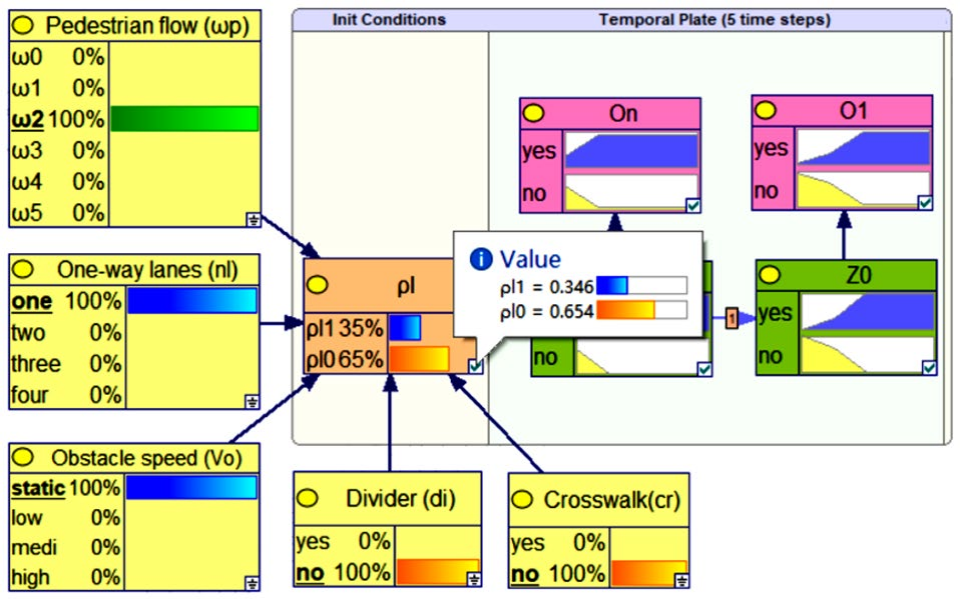
The prior probability $P(\rho_{l} = 1)$ reasoning (when $n_{l} = 2, \, di = 0, \, cr = 0, \, v_{o} = 0$).

The results show that the main factors affecting pedestrian "illegal crossing" are the number of lanes, traffic flow, vehicle speed, divider, and pedestrian crossing facilities and so on. In this paper, as show in Fig. 3, priori probability calculation model is established by considering environmental factors such as pedestrian flow ($\omega_{p}$), the number of one-way lanes($n_{l}$), divider($di$), crosswalk($cr$) and the speed of obstacles ($v_{o}$), i.e.:

3 $$ P(\rho_{l} = 1) = P(\rho_{l} = 1|cr,di,\omega_{p} ,v_{o} ,nl) $$

Table 1 details the meaning and status values of each node, which are described in detail in this part and “Posteriori probability of risk assessment”.

**Table 1:** The meaning and states of variables.

Node	State	Value	Meaning	Node	State	Value	Meaning
$cr$	Yes	1	Crosswalk	$v_{o}$	Static	0	$v_{o} = 0$ m/s
	No	0	Not crosswalk		Low	3	$0 < v_{o} \le 3$ m/s
$\omega_{p}$	$\omega 0$	0	$\omega = 0$(person time/h)		Medi	7	$3 < v_{o} \le 7$ m/s
	$\omega 1$	1	$0 < \omega /3600 \le 1$		High	9	$v_{o} > 7$ m/s
	$\omega 2$	2	$1 < \omega /3600 \le 2$	$di$	Yes	1	Have divider
	$\omega 3$	3	$2 < \omega /3600 \le 3$		No	0	No divider
	$\omega 4$	4	$3 < \omega /3600 \le 4$	$\rho_{l}$	*ρ*_*l*_1	1	Prior value of potential risk
	$\omega 5$	5	$\omega /3600 > 4$		*ρ*_*l*_ 0	0	
$n_{l}$	One	1	The number of one-way lanes	O	Yes	1	Observation values
	Two	2			No	0	
	Three	3		Z	Yes	1	Is there anyone on the road
	More	4			No	0	

It is not the focus of this paper that the value of the nodes of environmental impact factor should be acquired in the model. We assume that it can be accurately acquired through vehicle sensor and road network information. The probability distribution of $\rho_{l} = 1$ under the joint distribution of all environmental impact factors is conditional probability. Theoretical and practical studies show that the Poisson model can reasonably predict the probability of random events. Assuming that the number of pedestrians reaching the occluded areas follows the Poisson distribution:

4 $$ P(N(t) = n) = \frac{{(\lambda t)^{n} e^{ - \lambda t} }}{n!} $$

Suppose the average arrival rate of pedestrians per hour is $\omega$, we define $\omega_{p} = \omega /3600$, and propose the probability model of pedestrians crossing the road as follows:

5 $$ P(\rho_{l} = 1) = \lambda_{\rho } \cdot \left( {1 - P(N(t) = 0)) = \lambda_{\rho } \cdot (1 - e^{{ - \omega_{p} }} )} \right) $$

where $1 - e^{{ - \omega_{p} }} = 1 - P(N(t) = 0)$ represents the probability of pedestrians reaching the occluded area within 1 s,$\lambda_{\rho }$ is the environmental impact coefficient, indicating the impact of various environmental factors on pedestrian crossing the road.

6 $$ \lambda_{\rho } = \frac{{P_{{\text{c}}}^{(1 - cr)} \times K_{d}^{(di)} }}{{K_{v}^{{(v_{o} )}} \times n_{l} }} $$

where $P_{c}$ represents the probability of pedestrians crossing the street illegally without being affected by other environmental factors. $K_{d}$ and $K_{v}$ represent the "crosswalk influence coefficient" and "obstacle speed influence coefficient" respectively. $cr,di \in \left\{ {0,1} \right\}$ represent whether the pedestrian crossing area is a crosswalk and whether there is a divider in the center of the road respectively. $v_{o}$ represents the moving speed of the obstacle, and $n_{l} \in N*$ represents the number of one-way lanes. Based on some resemble research^37,38^, in this paper we set $P_{c} = 0.4$, $K_{d} = 0.36$, $K_{v} = 1.45$.

Bayesian reasoning is applicable to situations where the nature of nodes is discrete, so $\omega_{p}$ and $v_{o}$ is measured at the stage level. The values of various states are shown in Table 1. According to Eqs. (5) and (6), when $\omega_{p}$,$di$,$cr$,$v_{o}$ and $n_{l}$ are at different levels, $P(\rho_{l} = 1)$ has different values. Taking a non-signalized control section $n_{l} = 1$, $di = 0$, $cr = 0$,$v_{o} = 0$ as an example, $\omega$ detected by on-board sensor is $1 < \omega /3600 \le 2$, the probability $P(\rho_{l} = 1)$ reasoning result is 0.346, as shown in Fig. 3. Similarly, when $\omega_{p} = 0$, then $P(\rho_{l} = 1) = 0$; when $\omega_{p} = 5$, $n_{l} = 1$, $di = 0$, $cr = 1$,$v_{o} = 0$,then $P(\rho_{l} = 1) = 0.993 \approx 1$.

#### Posteriori probability of risk assessment $P(Z = 1|O)$

Taking $P(\rho_{l} )$ as a priori probability, the potential risk value of the AV passing through the interaction area is inferred according to Bayesian theory based on the observation data of the road section by the onboard sensor. $Z_{i}^{t} \in \{ 0,1\}$ is used to indicate whether there is TP on road cell $i$ at time $t$. $Z_{i}^{t} = 1$ indicates yes, and $Z_{i}^{t} = 0$ indicates no. Then, $P(Z_{i}^{t = 0} ) = P(\rho l)$, and the occupancy probability of road cell $i$ after $\Delta t$ sec is updated to $P(Z_{i}^{t + 1} )$. According to Markov property, there is:

7 $$ P(Z_{i}^{t + 1} |Z_{i + 1}^{t} ,Z_{i}^{t} ) = \sum\limits_{j = 0}^{1} {\sum\limits_{k = 0}^{1} {P(Z_{i + 1}^{t} = j)} } P(Z_{i}^{t} = k) \cdot {\rm K}_{z} $$

where ${\rm K}_{z}$ denotes the state transition matrix in the DBN, and its value can be allocated based on empirical data, in this paper we set $Z_{i - 1}^{t} = Z_{i}^{t - 1}$. As shown in Fig. 1, the road segment represented by the dotted line cells can be divided into two parts: the road cells observable by AV and those occluded by obstacle. According to Eq. (2), the left side of $x^{{t_{{}} }}$ is observable road cells, while the right side is occluded road cells. $O_{i}^{t} \in \{ 0,1\}$ is used to represent the observation result of the AV to cell $i$ at time $t$, where $O_{i}^{t} = 0$ indicates that no TP is observed on cell $i$, and $O_{i}^{t} = 1$ indicates that TP is observed on cell $i$. According to Bayesian theory, the occupancy probability of cell $i$ is inferred following observation as:

8 $$ P(Z_{i}^{t} = 1|O_{i}^{t} ) = \frac{{P(O_{i}^{t} |Z_{i}^{t} )P(Z_{i}^{t} = 1)}}{{P(O_{i}^{t} |Z_{i}^{t} = 0)P(Z_{i}^{t} = 0) + P(O_{i}^{t} |Z_{i}^{t} = 1)P(Z_{i}^{t} = 1)}} $$

where $P(O_{i}^{t} |Z_{i}^{t} = 1)$ represents the observed probability value when $Z_{i}^{t} = 1$. For visible road cells, the observation result is correlated with the road structure and performance of onboard visual equipment^15^. In the experiment, $P(O_{i}^{t} = 1|Z_{i}^{t} { = }1)$ = 0.9, $P(O_{i}^{t} = 1|Z_{i}^{t} { = }0)$ = 0.05, $P(Z_{i}^{t} )$ can be obtained by Eq. (7).

#### Risk distance coefficient

Most existing studies concerning the risk of collisions caused by occluded fields of view have regarded the potential virtual TPs within occluded areas as independent physical entities with motion functions only, which failed to consider their perceptual decision-making functions or their virtual interactions with AVs. In fact, regardless of whether they are pedestrians or vehicles, TPs usually pay attention to the surrounding environment when they entering an intersection area out from behind occluding object, especially at places with high traffic volumes. According to relevant research^4,15^, when the AV reaches the interaction area, the magnitude of risk with the magnitudes of the distance between the TP and the interaction area is nonlinearly correlated because most TPs are also agents with the ability to adjust their states. At larger distances, TPs have more time to judge the dangers and make timely adjustments to speed or direction, thereby reducing the risk. In contrast, a smaller distance indicates greater risk.

**Figure 4:**
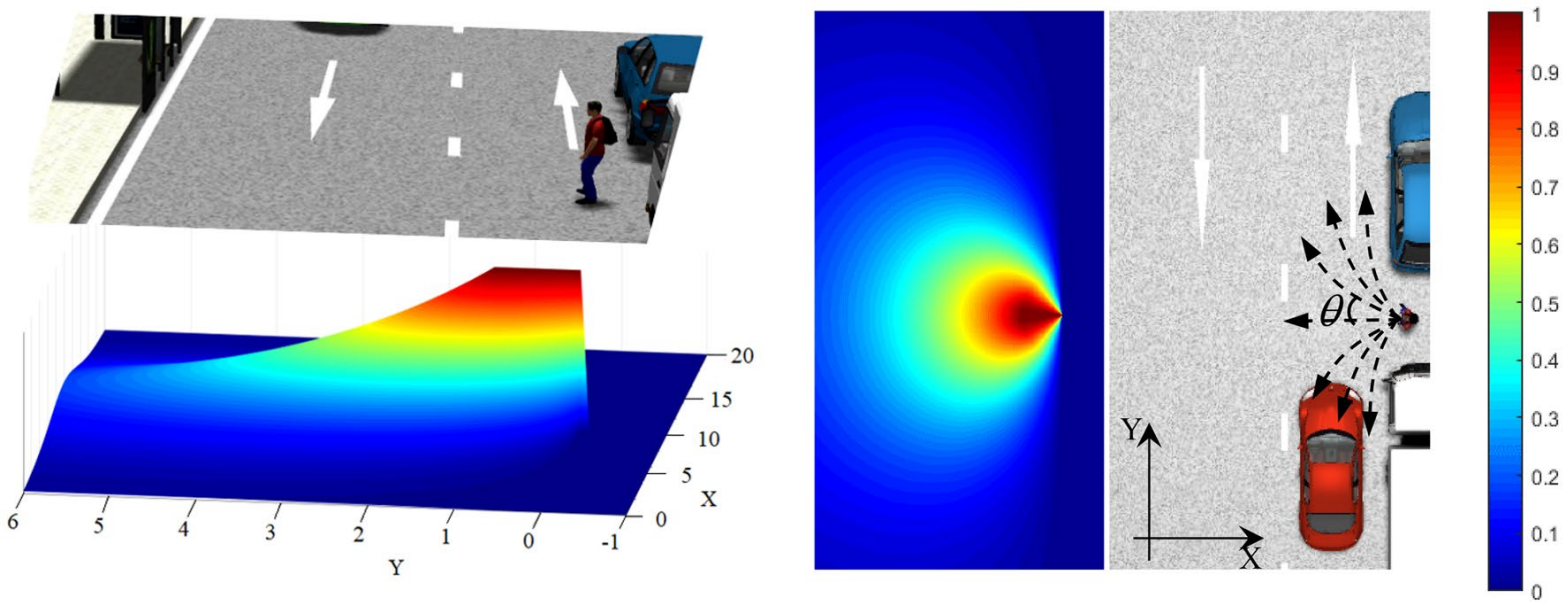
Distance coefficient field graph $k = 1$.

McGill was developed a "conditional risk" model integrating drivers' attention, which describes the nonlinear relationship of vehicle distance from intersection areas with risk^15^. Actually, the magnitude of potential risks is linked not only to the pedestrian speed and the distance between TPs and AVs but also to factors such as speed direction and perceptual capability of TPs. On the basis of the "conditional risk" model, this paper proposes a risk function that integrates the attention, speed, and perceptual interaction capabilities of TPs, which is called the risk distance coefficient and denoted by $K_{d}$.

9 $$ K_{d} = \left\{ {\begin{array}{*{20}l} 1 \hfill & {d_{i} < d_{s} } \hfill \\ {\exp \left[ { - \lambda_{d} \frac{{k(d_{i} - d_{s} )}}{{\sigma_{d}^{2} }}} \right] \, \cdot |\cos (\theta )|{ (}{\pi \mathord{\left/ {\vphantom {\pi 2}} \right. \kern-\nulldelimiterspace} 2} \le \theta \le {{3\pi } \mathord{\left/ {\vphantom {{3\pi } 2}} \right. \kern-\nulldelimiterspace} 2}{)}} \hfill & {otherwise} \hfill \\ \end{array} } \right. $$

where $d_{i}$ represents the vertical distance from the TP on cell $i$ to the intersection area. $d_{s}$ is the minimum safe distance, in the case of pedestrians, $d_{s} = 0.8$ m in the experimentation; $\sigma_{d}$ is the standard deviation. Parameter $0 \le \lambda_{d} \le 1$ indicates the attention degree of TPs in the model to the surrounding environment, parameter $k \in \left\{ {0,1} \right\}$ indicates the perceptual capability of TPs. $k = 0$ indicates perceptually incapable, such as children or moving objects, and $k = 1$ indicates perceptually capable. In addition, $\theta$ is defined as the angle between the movement direction of TPs and the line connecting the intersection area and TPs, ${\pi \mathord{\left/ {\vphantom {\pi 2}} \right. \kern-\nulldelimiterspace} 2} \le \theta \le 3{\pi \mathord{\left/ {\vphantom {\pi 2}} \right. \kern-\nulldelimiterspace} 2} \,$. Figure 4 depicts the risk distance coefficient between the pedestrian and the intersection area.

If the time taken for pedestrians to arrive at the intersection area is longer than for the vehicle to pass through the intersection area, i.e., at farther distances, the area can be regarded as less dangerous. When the distance from pedestrians to the intersection area is less than the safe distance $d_{s}$, the risk distance coefficient $K_{d} = 1$.

#### Potential risk assessment

Finally, the potential risk on road cell $i$ is constituted by the product of $P(Z_{i}^{t} = 1|O_{i}^{t} )$ and the $K_{d}$, which can be obtained by Eqs. (8) and (9) as:

10 $$ \gamma_{c,i}^{t} = K_{d} \cdot P(Z_{i}^{t} = 1|O_{i}^{t} ) \, (i \in [m^{t} ,n^{t} ]) $$

By substituting Eqs. (10) into (1), the potential risk arising from the visually occluded area is precisely obtained.

$$ \gamma_{c} = \mathop {\arg \max }\limits_{{\gamma_{c,i}^{t} }} \{ {\mathbb{R}}[\gamma_{c,i}^{t} \, |O^{t} ] \, (i \in [m^{t} ,n^{t} ])\} = \arg \max \{ K_{d} \cdot P(Z_{i}^{t} |O_{i}^{t} ) \, (i \in [m^{t} ,n^{t} ])\} $$

The observation result $O_{i}^{t}$ of cell $i$ is a time-dependent discrete value. The model performs sampling and computation at time intervals of $\Delta t$. The potential risk assessment algorithm for occluded areas is shown in Algorithm I.
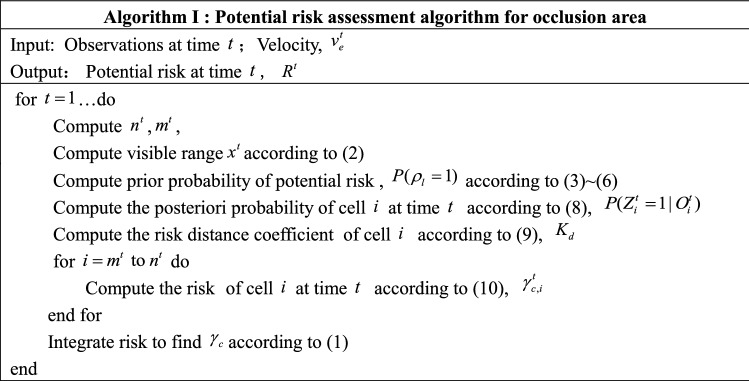


### Application of the model

Based on the above risk assessment model, this section applies the model to the motion planning of AVs under occluded vision for safe driving. Inspired by the work of Yu et al.^14^, we use $\gamma_{c}$ to adjust the speed and heading angle in the AV motion planning, which can improve the safety and comfort of the AV. Our purpose is to ensure the safety of AV operation and improve passenger comfort by changing the speed and heading angle according to the potential risk.

#### Vehicle speed

When there is no risk, or the risk is less than the threshold $\, \gamma_{go}$, the desired vehicle speed $v_{e}^{des} \,$ is set as the speed limit $v_{l} \,$ of the current road section. When the potential risk is predicted, the desired vehicle speed should be adjusted, we set the desired speed as:

11 $$ v_{e}^{des} { = }v_{l} { (1 - }\gamma_{c} {)} $$

##### Deceleration

At time $t$, the AV runs on the predetermined route at the speed of $v_{e}^{t}$, due to the obstruction of the front vision, the AV starts the potential risk assessment model. When the vehicle observes danger or the sudden emergence of pedestrians, first consider the safety and decelerate with the deceleration $a_{e}^{ - }$. The greater the absolute value of $a_{e}^{ - }$, the shorter the braking distance, and the higher safety, but the lower the comfort, the vice versa. In motion planning, the security cost $L_{saf} (a_{e}^{ - } )$ associated with $a_{e}^{ - }$ should be considered first as:

12 $$ L_{saf} (a_{e}^{ - } ) = \exp \left\{ {\frac{{ - (s_{{a_{e}^{ - } }} - s_{{a_{e,\max }^{ - } }} )^{2} }}{{\sigma_{saf}^{2} }}} \right\} = \exp \left\{ {\frac{{ - \frac{1}{4}[(a_{e}^{ - } - a_{e,\max }^{ - } ) \cdot t_{a}^{2} ]^{2} }}{{\sigma_{saf}^{2} }}} \right\} $$

where $a_{e,\max }^{ - }$ represents the maximum deceleration in the AV performance index. When the AV decelerates with $a_{e,\max }^{ - }$, the braking distance is the shortest and the lowest safety cost. $\sigma_{saf}$ is the standard deviation at which the nonlinear least-squares optimization method can be solved based on the collected driving experience data.$s_{{a_{e}^{ - } }}$ and $s_{{a_{e,\max }^{ - } }}$ are represent the forward distance of the vehicle under deceleration $a_{e}^{ - }$ and $a_{e,\max }^{ - }$ in unit time $t_{a}$ respectively. When the deceleration is $a$.

13 $$ s_{a} = v_{e}^{t} \cdot t_{a} - \frac{{a \cdot t_{a}^{2} }}{2} $$

In addition to the safety cost $L_{saf} (a_{e}^{ - } )$, the comfort cost $L_{com} (a_{e}^{ - } )$ is also considered to drive the AV to meet the required speed $v_{e}^{t}$.

14 $$ L_{com} (a_{e}^{ - } ) = \exp \left\{ {\frac{{ - (\Delta v_{{a_{e}^{ - } }} - \Delta v_{{a_{e,\min }^{ - } }} )^{2} }}{{\sigma_{com}^{2} }}} \right\} = \exp \left\{ {\frac{{ - [(a_{e}^{ - } - a_{e,\min }^{ - } ) \cdot t_{a} ]^{2} }}{{\sigma_{com}^{2} }}} \right\} $$

where $\Delta v$ represents the speed change in unit time $t_{a}$, $a_{e,\min }^{ - }$ is the minimum deceleration (the highest comfort), and $\sigma_{com}$ is the standard deviation. According to Eq. (12) and Eq. (14), the optimal deceleration threshold $a_{e}^{ - }$ during braking can be found by solving the optimization problem of Eq. (15):

15 $$ \begin{gathered} \mathop {\min }\limits_{{a_{e}^{ - } }} \, \lambda^{ - } \cdot L_{saf} (a_{e}^{ - } ) + (1 - \lambda^{ - } )L_{{{\text{co}} m}} (a_{e}^{ - } ) \hfill \\ s.t. \, v_{e,\min }^{{}} \le v_{e}^{t} + a_{e} \cdot t_{a} \le v_{e,\max } {; }|a_{e,\min }^{ - } | \, \le \, |a_{e}^{ - } | \, \le \, |a_{e,\max }^{ - } | \hfill \\ \end{gathered} $$

where $v_{e,\min }^{{}}$ and $v_{e,\max }^{{}}$ are the minimum speed and the maximum speed of the AV respectively, and the values can be seen in Table 2. $\lambda^{ - }$ represents the security cost weight, and a larger $\lambda^{ - }$ means that the vehicle has higher requirements for safety. Passing on $\lambda^{ - }$, the “maximum comfortable deceleration”$a_{e}^{ - }$ can be obtained, represents the maximum deceleration selected by the AV in the view of comfort properties. When the real-time speed $v_{e}^{t} \,$ of the AV exceeds the expected speed $v_{e}^{des} \,$ or there is a potential danger, the vehicle will adjust the speed in real-time according to the distance $s_{e}^{t}$ and the speed $v_{e}^{t}$, $v_{e}^{t + 1} = v_{e}^{t} - a_{e}^{t} \cdot dt \,$, where the real-time deceleration $a_{e}^{t}$ is:

16 $$ a_{e}^{t} = {{\left( {v_{e}^{t} } \right)^{2} } \mathord{\left/ {\vphantom {{\left( {v_{e}^{t} } \right)^{2} } {2s_{e}^{t} }}} \right. \kern-\nulldelimiterspace} {2s_{e}^{t} }}\quad \, s.t.{ | }a_{e}^{t} | \, \le \, |a_{e}^{ - } | \, \le \, |a_{e,\max }^{ - } | $$

**Table 2:** Parameters for simulations.

(Equation) Parameter	Value
$\Delta t$, $v_{p}$	0.1 s, 1.5 m/s or 2.0 m/s
(2) $y_{p}$,$d_{o}$	1.5–2.0, 2.0–5.0 m
(4) $P_{c}$,$K_{d}$,$K_{v}$	0.40, 0.36, 1.45
(7) $K_{z}$	$Z_{i + 1}^{t} = Z_{i}^{t - 1}$
(9) $d_{s}$,$\sigma_{d}$,$\lambda_{d}$	0.8 m, 4.7, 0.9
(12) $\sigma_{saf}$ (14) $\sigma_{com}$	1.05, 1.5,
(12) $a_{e,\min }^{ - }$,$a_{e,\max }^{ - }$	0 m/s^2^, − 6 m/s^2^
(17) $a_{e,\min }^{ + }$,$a_{e,\max }^{ + }$	0 m/s^2^, + 6 m/s^2^
(15) $\lambda^{ - }$,(19)$\lambda^{ + }$	0.9, 0.25
(20) $\gamma_{{{\text{go}}}}$	$P(Z = 1|O = 0)$
(21) $\tau$	0.2 s
(22) $\sigma_{e}$	4.9
(24) $a_{thresh}$	4 m/s^2^

##### Acceleration

Acceleration operation is generally operated when there is no risk or the risk is low, so the acceleration planning in this study mainly considers the comfort and conservatism of vehicle driving. The calculation model of acceleration is similar to the calculation of deceleration, which only needs to change the weight (use $\lambda^{ + }$ represents conservative cost weight). The comfortable acceleration $a_{e}^{ + }$ during acceleration can be obtained by reducing the cost weight of conservative, the specific description is shown in Eqs. (17–19).

17 $$ L_{saf} (a_{e}^{ + } ) = \exp \left\{ {\frac{{ - \frac{1}{4}[(a_{e}^{ + } - a_{e,\max }^{ + } ) \cdot t_{a}^{2} ]^{2} }}{{\sigma_{saf}^{2} }}} \right\} $$

18 $$ L_{com} (a_{e}^{ + } ) = \exp \left\{ {\frac{{ - [(a_{e}^{ + } - a_{e,\min }^{ + } ) \cdot t_{a} ]^{2} }}{{\sigma_{com}^{2} }}} \right\} $$

and

19 $$ \begin{gathered} \, \mathop {\min }\limits_{{a_{e}^{ + } }} \, \lambda^{ + } \cdot L_{saf} (a_{e}^{ + } ) + (1 - \lambda^{ + } )L_{{{\text{co}} m}} (a_{e}^{ + } ) \hfill \\ s.t. \, v_{e,\min }^{{}} \le v_{e}^{t} + a_{e} \cdot t_{a} \le v_{e,\max } { ; }a_{e,\min }^{ + } \le \, a_{e}^{ + } \le \, a_{e,\max }^{ + } \hfill \\ \end{gathered} $$

Based on the foregoing analysis, the speed of AVs is formulated as follows:

20 $$ v_{e}^{t + 1} = \left\{ {\begin{array}{*{20}l} {v_{e}^{t} - a_{e}^{t} \cdot dt \, v_{e}^{t} > v_{e}^{des} } \hfill & {{\text{or (}}\gamma_{c} { > }\gamma_{{{\text{go}}}} {\text{ and }}d_{e}^{t} \ge s_{e}^{t} {)}} \hfill \\ 0 \hfill & {\gamma_{c} \ge \gamma_{{{\text{go}}}} {\text{ and }}s_{e}^{t} { = }0} \hfill \\ {\min (v_{e}^{des} ,v_{e}^{t} + a_{e}^{ + } \cdot dt)} \hfill & {v_{e}^{t} < v_{e}^{des} {\text{ and }}\gamma_{c} { < }\gamma_{{{\text{go}}}} } \hfill \\ {v_{e}^{t} } \hfill & {{\text{otherwise}}} \hfill \\ \end{array} } \right. $$

where $d_{e}^{t}$ represents the braking distance of the AV at $v_{e}^{t}$ speed and acceleration $a_{e}^{t}$:

21 $$ d_{e}^{t} = v_{e}^{t} \cdot \tau + \frac{{v_{e}^{t2} }}{{2a_{e}^{t} }} $$

where $\tau$ represents the vehicle operation delay time, which in this paper is $\tau = 0.2$ seconds.

#### Heading angle

According to Eq. (2), the farther the lateral distance between the vehicle and the occluded area, the more conducive it is to increasing visibility and reducing risk, therefore, change the heading angle $\theta_{ego}$ when the AV is running to achieve lateral motion control. Inspired by the potential field method, a threat model of potential risk in the occluded area to surrounding vehicles was established as:

22 $$ \Gamma_{e} = \gamma_{c} .\exp \left( { - \frac{{d_{eo}^{2} }}{{2\sigma_{e}^{2} }}} \right) $$

where $0 \le \Gamma_{e} \le \gamma_{{\text{c}}}$, indicates the threat degree of potential risk to AV. $\sigma_{e}$ is the standard deviation of the potential field, and $d_{eo}$ is the distance between the AV and the edge of the occluded area (the junction of the visible area and occluded area). By mapping risk $\gamma_{c}$ to the threat degree $\Gamma_{e}$ through Eq. (22), the vehicle's heading is deflected $\theta_{ego}$:

23 $$ \theta_{ego} = \pm \lambda_{e} \cdot \arcsin \Gamma_{e} $$

where $\lambda_{e}$ is the risk repulsion factor used to optimize $\theta_{ego}$. “$\pm$” indicates left or right deflection. In part V, this paper takes $\lambda_{e}$ = 1. When the AV is allowed to lateral movement, it is possible to reduces the risk or increases visibility by lateral motion. According to Eq. (23), $\theta_{ego}$ is inversely proportional to $d_{eo}$, and real-time risk $\gamma_{c}$ is in direct proportion. Similarly, after the vehicle bypasses the occluded areas, it takes the initially planned target route as the final attraction target and horizontally returns to the target route.

The potential risk assessment model-based velocity planning algorithm for intermittently occluded areas is shown in Algorithm II.
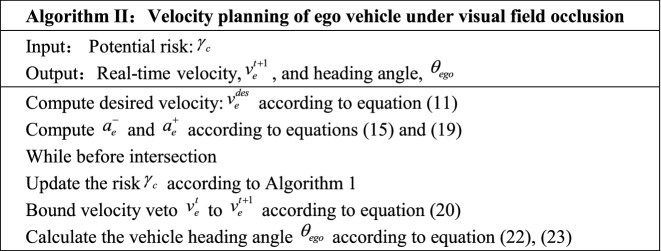


### Validation

To validate the effectiveness of the proposed model, a dynamic simulation model was built based on Python programming, with which simulation verification is performed by setting up two typical occluded vision scenes based on the natural environment. Furthermore, comparison was made with other existing methods. The values of relevant parameters are shown in Table 2, in this paper, the meter is the unit of length and the second is the unit of time.

### Scene setting

#### Straight road scene

**Figure 5:**
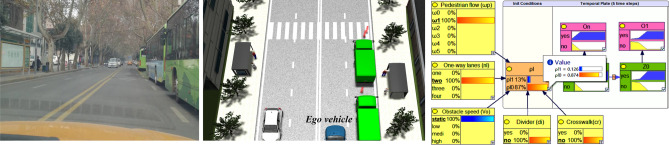
On the left-hand side, shows the actual scene where pedestrians are easy to darting out. On the right-hand side, shows the reasoning results of priori probability using our method.

The straight road scene selects the road section with continuous parking in the city, as shown in the picture on the left of Fig. 5, three buses are parking continuously at the bus stop, and there is another bus stop on the opposite of the road. In this scenario, there is a high probability for pedestrians to darting out to the opposite bus stop. The speed limit of the road section is $v_{l}$ = 36 km/h (10 m/s), the number of one-way lanes is 2, there is no divider, and the flow of people is $0 \le \omega /3600 \le 1$ person time/hour. According to Eqs. (5) and (8): when $cr = 0$, then $P(\rho l = 1)$ = 0.126, $P(Z = 1|O = 0)$ = 0.015, $P(Z = 1|O = 1)$ = 0.722, as shown in the picture on the right of Fig. 5, when $cr = 1$, then $P(\rho l = 1)$ = 0.316, $P(Z = 1|O = 0)$ = 0.046, $P(Z = 1|O = 1)$ = 0.893.

**Figure 6:**
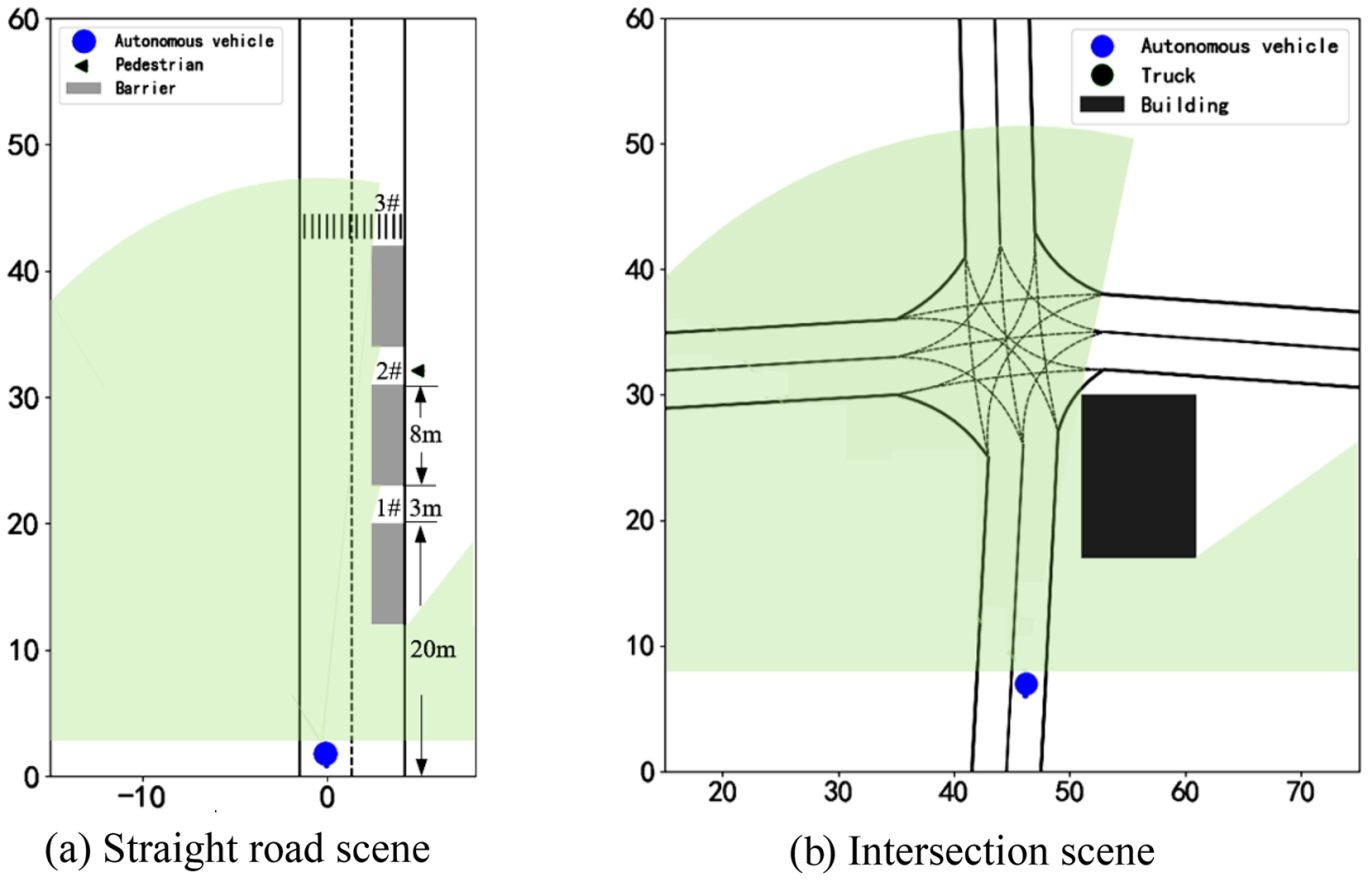
Simulation test scenario structure.

In Fig. 5, the length of the test road we selected is 60 m, and the road structure is shown in Fig. 6a. There are three occluded areas (1#, 2#, 3#) on the right side of the road. The situation of pedestrians crossing the road randomly in the occluded area, and to verify the model, we simulated the random pedestrian model by uses random numbers simulation in the experiment.

#### Intersection scene

To further validate the effectiveness of the proposed method, we verified the left turn experiment of AVs at intersections without signal lights, as shown in Fig. 6b, in this traffic scene we set $n_{l} = 2$ $n_{l} = 2$, $di = 0$,$cr = 1$, $v_{o} = 0$, $\omega_{p} = 1$. We recreated the scene for verification by using the same map, initial speed of 9 m/s, expected speed of 9 m/s, maximum acceleration, and minimum acceleration provided in the some works^14,21^, the traffic participants are pedestrians and other vehicles on the road, and their maximum speed is set to 10 m/s. To simulate scenarios with heavy occlusion, the occlusions added in this scene are the building which in roadside and bulky vehicles running on roads.

## Results and analysis

### Straight road scene

**Figure 7:**
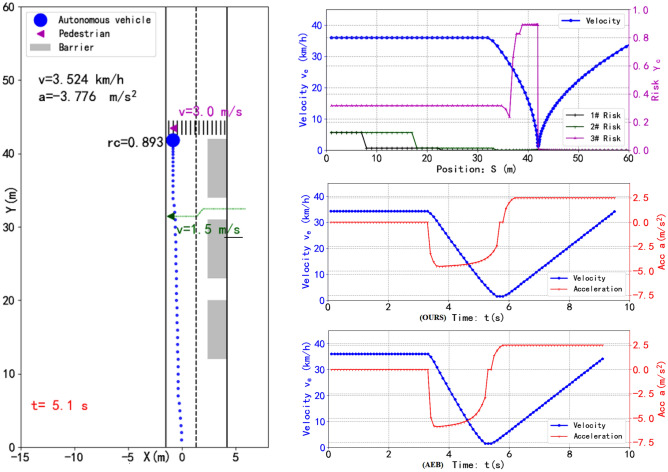
The screenshot of the dynamic simulation test is on the left, and the top and middle graph of the right is the position–velocity, risk curve, and the time–velocity, acceleration curve respectively, which are generated by the proposed method. The bottom graph on the right shows the velocity and acceleration curves generated by the AEB method.

In all simulation test, there was no collision occurred. Even if there is no sudden pedestrian, the AV will be moderately slow down at high speed and be as far away from the risk area as possible laterally to increase the visual range and reduce the risk, as shown in Fig. 7. Figure 7 describes only one type case.

When AV passes through the occluded area 1#. When pedestrians suddenly rush out, the vehicle can also slow down or stop smoothly without fierce shaking to avoid accidents, and performs well in ride comfort. In area 2#, the pedestrian changes his direction for safety (as shown in the green track), so the AV keeps moving at a constant speed without slowing down. Compared with the profiles generated by AEB, that is adopted in the works of Schratter et al.^29^, the AV can safely also pass through the risk areas with the distance is 60 m using the proposed method. Experimental in the same environment showed the AV’s acceleration of our method is generally lower and the AV is more comfortable.

However, to the best of our knowledge, there is no common computational metric in the literature for ride comfort. We using the following Discomfort Score ($DS$), which defined by Yu et al.^14^ to represent a continuous range of discomfort:

21 $$ DS = \frac{1}{T}\int\limits_{0}^{T} {\max \left( {0,\left| {a_{e}^{t} } \right| - a_{thresh} } \right)dt} $$

where $T$ is the duration to reach the goal, and $a_{thresh}$ is the threshold of comfortable deceleration. In the same situation under this scene as show in Fig. 7, when the AV crosses the 60 m test road, the AEB method $DS$ = 2.67, taking 9.0 s, and the proposed method $DS$ = 0.63, taking 9.5 s. But when no pedestrian darting out, both methods are the same in terms of $DS$ and time consuming. Due to the randomness of pedestrian crossing the street illegally, we simulated 1000 cases of pedestrian crossing randomly when AV adopted two methods to pass through the scene shown in Fig. 6a. The average discomfort score, $DS_{aver}$, and the average time consuming, $T_{aver}$, as follows:

$$ {\mathbf{AEB}}:DS_{aver} = 2.14,\,T_{aver} = 7.9 \, s; $$

$$ {\text{The}}\,{\text{proposed}}\,{\text{method}}:DS_{aver} = 1.02,T_{aver} = 9.1 \, s. $$

The result shows that, when facing the scene with occluded area, using our method will make the ego-vehicle behavior more cautious. In the emergency of darting out of TPs, the proposed method makes the ego-vehicle more preventive and comfortable than the method of AEB.

### Intersection scene

**Figure 8:**
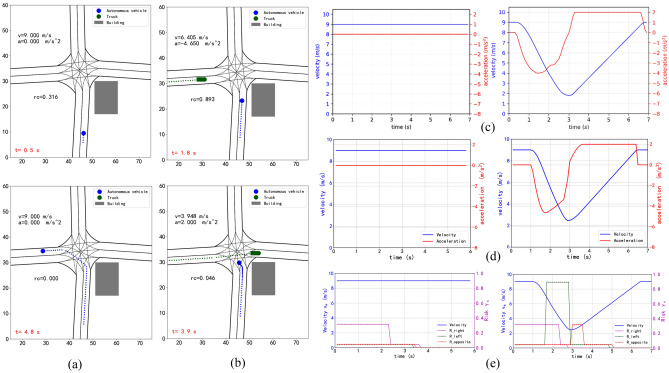
(**a**) Scenario of intersection with one static occlusion (building) and no incoming traffic. (**b**) The same scenario with one other vehicle (truck) coming from the left. (**c**) Speed and acceleration profiles obtained by Yu et al.^14^ in two scenarios (the left one generated by scenario (**a**), the right one generated by scenario (**b**)). (**d**) Speed and acceleration profiles of ours. (**e**) Speed and risk profiles of ours.

Figure 8c, d describe the speed and acceleration profiles generated by using the ethod proposed by Yu et al.^14^ and using our proposed method to realize the left turn of the AV in two cases respectively. Figure 8e shows various potential risk curves predicted by the AV using the method proposed in this paper in scenarios (a) and (b) respectively. In the figure, “$R - right$” is the potential risk arising from the right road at crossroad which occluded by the building, “$R - left$” is the potential risk arising from the left road at crossroad, “$R - opposite$” is the potential risk arising from the oncoming lane while AV crossing the intersection, they are represent the risk probability of collision between the AV and the other vehicles if AV's speed adjustment is not made. Both the method of Yu et al.^14^ and ours are make the AV turn left safely without collision, for two scenarios, our method has obtained the speed profile which is basically consistent with the speed profile obtained by Yu et al.^14^.

For the first scenario, our method obtains zero acceleration, this is because the initial speed of the vehicle is low. When the initial speed of the AV was increased from 9 to 11 m/s, the test found that the speed of the forward movement of AV would drop somewhat, this due to the potential risk of the road blocked by the building is detected by AV. Under the condition of higher speed, the AV will appear more cautious, which is more in line with natural human driving habits, a cautious approach is reasonable when the full state of the world cannot be observed. So, our method is also suitable for similar intersection scenes with occlusions.

**Figure 9:**
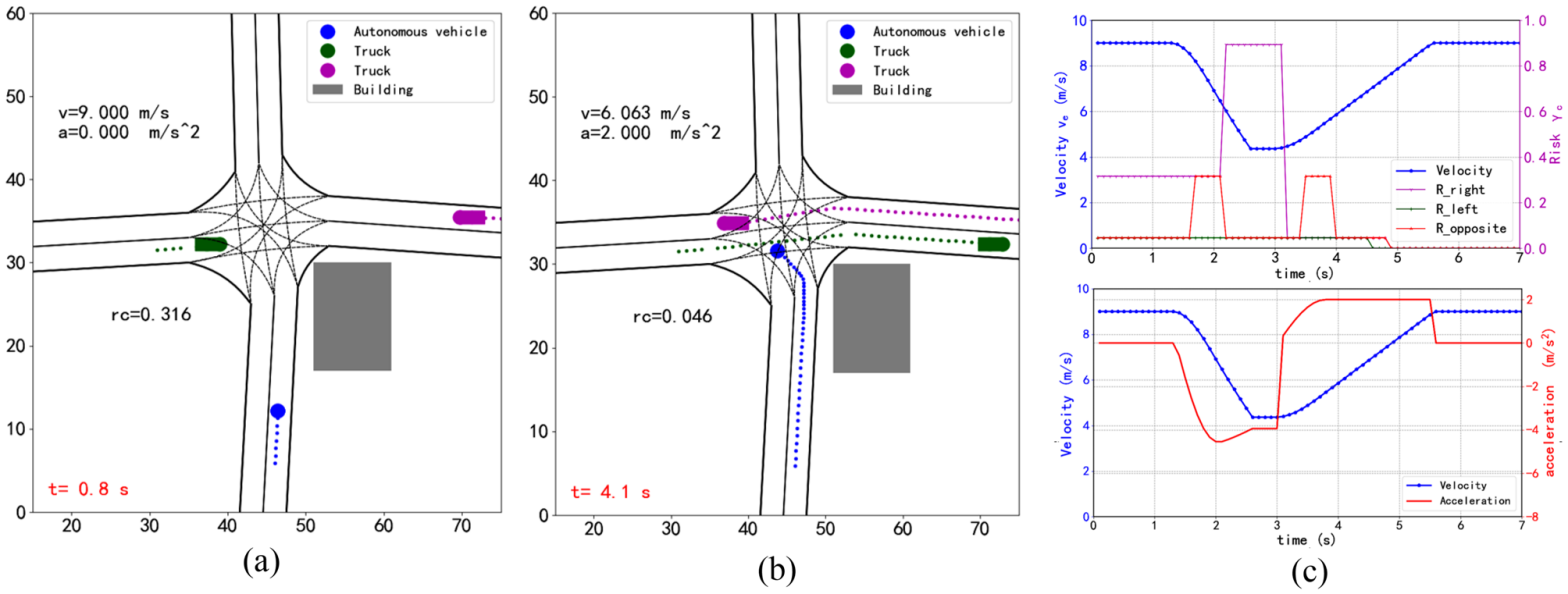
(**a**), (**b**) Scenario of intersection with one other vehicle (truck) coming from the left road at crossroad and one other vehicle (truck) coming from the right road which occluded by the building. (**c**) Speed and risk profiles of AV.

In order to verify that our method is applicable to traffic scenes with visual occlusion, we add traffic vehicles in the Fig. 9 scenario. When the other vehicle suddenly appears from the area which occluded by the building, the AV can observe the surrounding environmental factors, change the speed according to the potential risks of reasoning from all directions, and complete the turn left safely without collision, as show in Fig. 9a and Fig. 9b. There are two fluctuations in the “$R - opposite$” in Fig. 9c, which are represent the potential risks of the oncoming lane being occluded by the two vehicles when they crossing the intersection.

With good generalization adaptability, our method is suitable for most traffic scenes with visual occlusion. By verification, this method is suitable for scenes on road and intersection with single occlusion or intermittent occlusion, such as pedestrian crossings, bus stops and intersections. Complex scenes in real traffic, such as roundabout or suburban roads, can be decomposed into several road or intersection with small curvature. In addition, since the proposed method is inseparable from the observation of the surroundings by on-board sensors, if the weather is bad, such as fog or night with low visibility, our work may face a challenge, which is also a problem that we need to solve in our future research. For more complex and special scenes, we will explore more factors affecting potential risks.

## Conclusion

This paper proposed a potential risk assessment model for AVs under occluded vision. Utilizing Bayesian theory, the model can infer the potential risks arising from TPs in occluded areas based on factors such as the surrounding environment of the occluded area, thereby allowing AVs to safely and efficiently pass through the dangerous interactive areas under limited perceptual data. Under the assumption of the AV’s travel path has been planned, from the security, comfort and careful driving aspects, the AV movement was improved by the speed and heading angle control. Through the simulation test, the presented approach allows AV advancing into the road in a comfortably and cautious manner, successively increasing the observable area. The model is suitable for scenes with intermittent occlusion, and also applicable to scene of intersection without signal light and visual field occlusion. However, since the proposed method is inseparable from the observation of the surroundings by on-board sensors, if the weather is bad, such as fog or night with low visibility, our work may face a challenge, which is also a problem that we need to solve in our future research. For more complex and special scenes, we will explore more factors affecting potential risks. In addition, the environmental factors considered in the model are not comprehensive enough, especially when there are many dynamic obstacles, the model needs to be further optimized. On the basis of the current work, we will conduct future research in the following three directions:

1. Further study the potential risk assessment of complex traffic scenes, especially with many dynamic obstructions.
2. Combined with the road risk analysis of traffic engineering, we will systematically establish a collision risk analysis (DBN inference) model under occluded vision and integrate the established risk assessment model into the complete motion planning method of AVs.
3. Explore more factors affecting the potential risk and discuss how to adjust the road structure and natural data used in the model and learn the best model parameters.
